# Osmolality‐Independent Impact of Sodium on Glycosylation of an Fc‐Fusion Protein and the Hexosamine Biosynthesis Pathway in a Chinese Hamster Ovary Cell Line

**DOI:** 10.1002/biot.70261

**Published:** 2026-06-17

**Authors:** Jason Reiniger, Adrian Pöttner, Lara Rosenberger, Aline Zimmer

**Affiliations:** ^1^ Merck Life Science KGaA Upstream R&D Darmstadt Germany; ^2^ Institute For Organic Chemistry and Biochemistry Technische Universität Darmstadt Darmstadt Germany; ^3^ Merck KGaA Analytical Department Darmstadt Germany

**Keywords:** cell culture medium, Fc‐fusion protein, glycosylation, hexosamine biosynthesis pathway, osmolality, sodium chloride

## Abstract

This study investigates the osmolality‐independent effects of sodium and potassium on the glycosylation of an Fc‐fusion protein and the activity of the hexosamine biosynthesis pathway in a Chinese Hamster Ovary cell line. Previous research linked low molecular weight proteoforms of this Fc‐fusion protein to reduced *N*‐glycan complexity and *O*‐glycan site occupancy. Through a series of batch experiments, we demonstrated that increased concentrations of sodium or potassium ions led to a reduction of these proteoforms. Our findings suggest that ion availability impacts the activity of the hexosamine biosynthesis pathway, thereby enhancing the availability of uridine diphosphate *N*‐acetylglucosamine, which is a crucial substrate for glycosylation. Notably, these changes in nucleotide sugar concentration were independent of the increased osmolality. Through supplementation of intermediates that are funneled into the hexosamine biosynthesis pathway, a link between Fc‐fusion protein quality and activated sugar availability was established as each supplement that elevated nucleotide sugar concentrations reduced low molecular weight proteoforms. We hypothesize that changes in sodium and potassium concentrations lead to increased uptake of nutrients and calcium, influencing metabolic pathways and enzyme activity. Altogether, this work highlights the importance of ion balance in cell culture media development for optimizing correct glycosylation during therapeutic protein production.

## Graphical Abstract and Lay Summary

Sodium and potassium supplementation in CHO batch culture elevates intracellular UDP‐HexNAc concentrations through activation of the hexosamine biosynthesis pathway (HBP), independently of osmolality. Increased nucleotide sugar availability enhances Fc‐fusion protein glycosylation and reduces the abundance of low molecular weight proteoforms. Direct HBP substrate supplementation (glutamine & glucosamine) confirms the link between activated sugar availability and improved protein glycosylation quality.

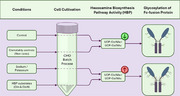

**Abbreviations**
CCM: Cell culture medium
CHO: Chinese hamster ovary
Ectoine: (4S)‐2‐Methyl‐3,4,5,6‐tetrahydropyrimidine‐4‐carboxylic acid
Fc: Fragment crystallizable
FDA: U.S. Food and Drug Administration
GFAT: Glutamine‐fructose‐6‐phosphate transaminase
GlcN: Glucosamine
GlcNAc: *N*‐acetylglucosamine
Gln: Glutamine
GS: Glutamine synthetase
HBP: Hexosamine biosynthesis pathway
HMW: High molecular weight
LMW: Low molecular weight
mAb: Monoclonal antibody
Na:K ratio: Sodium‐to‐potassium ratio
PFK: Phosphofructokinase
SEC: Size exclusion chromatography
UDP‐GalNAc: Uridine diphosphate N‐acetylgalactosamine
UDP‐GlcNAc: Uridine diphosphate N‐acetylglucosamine
VCD: Viable cell density

## 1 Introduction

Antibody‐based therapeutics represent an important class of pharmaceuticals, with a total of 220 products approved by the U.S. Food and Drug Administration (FDA) between 1986 and the end of 2025 [1, 2, 3]. Their relevance in the pharmaceutical market is highlighted by a worldwide value increase of $202 billion (USD) over the past decade [3, 4]. In addition to mAbs, Fc‐fusion proteins represent another important subclass of antibody‐based therapeutics. Among the 220 antibody products approved by the FDA, 19 were Fc‐fusion proteins [1, 3]. Fc‐fusion proteins are engineered by fusing a protein that specifically binds an antigen with the fragment crystallizable (Fc) part of an Immunoglobulin G. The Fc part enhances both pharmacokinetic and pharmacodynamic properties while also simplifying the purification process. The antigen‐binding domain varies depending on the specific application of the biotherapeutic [5]. One historically successful Fc‐fusion protein is Etanercept (Enbrel, Pfizer/Amgen), which was approved by the FDA in 1998 for the treatment of rheumatoid arthritis. Etanercept consists of the extracellular domain of human p75 tumor necrosis factor receptor fused with a human Immunoglobulin G1 Fc domain. It neutralizes both soluble and membrane‐bound tumor necrosis factor, thereby reducing inflammatory effects [6]. More recently, two Fc‐fusion proteins were approved by the FDA in 2024: Nogapendekin alfa inbakicept (Anktiva, ImmunityBio) and Sotatercept (Winrevair, Merck). Nogapendekin alfa inbakicept is a protein complex, where the N‐terminal region of the Interleukin‐15 receptor‐α is fused to the Fc dimer of Immunoglobulin G1. Anktiva was approved for the treatment of bladder cancer. Sotatercept is a human activin receptor type 2A Fc‐fusion protein designed for the treatment of pulmonary arterial hypertension [3].

**FIGURE 1:**
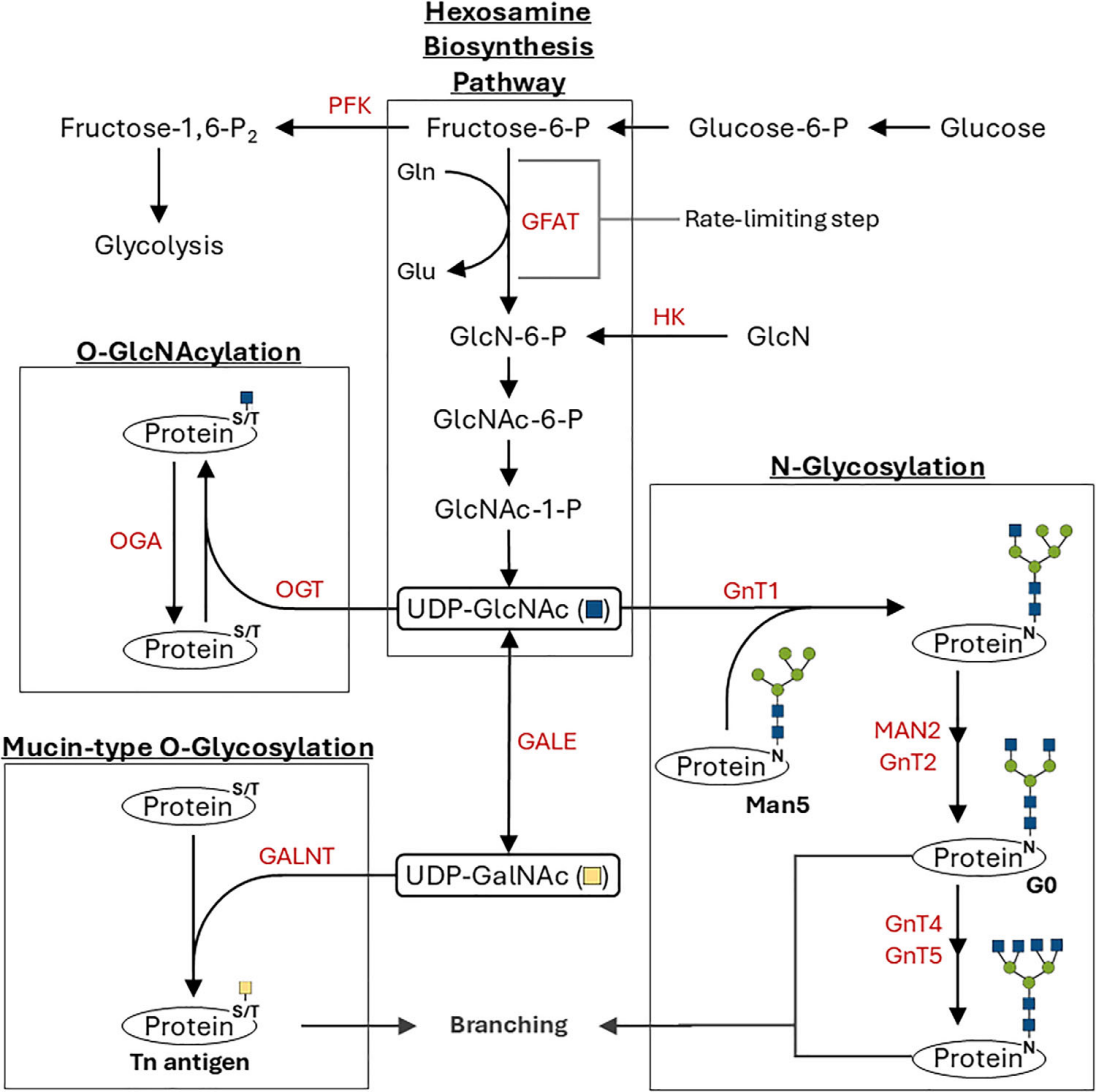
Overview of the hexosamine biosynthesis pathway (HBP) and associated glycosylation processes. In the first and rate‐limiting step of the HBP, fructose‐6‐phosphate (Fructose‐6‐P) and glutamine (Gln) are converted to glucosamine‐6‐phosphate (GlcN‐6‐P) by glutamine‐fructose‐6‐phosphate transaminase (GFAT). Uridine diphosphate *N*‐acetylglucosamine (UDP‐GlcNAc) is the end product of the HBP and is an important substrate in *N*‐glycosylation and *O*‐GlcNAcylation. UDP‐GlcNAc is also converted to uridine diphosphate *N*‐acetylgalactosamine (UDP‐GalNAc) which is the substrate required for the initial step in mucin‐type *O*‐glycosylation. Fructose‐1,6‐P_2_, fructose‐1,6‐bisphosphate; Fructose‐6‐P, fructose‐6‐phosphate; GALE, UDP‐glucose‐4‐epimerase; GALNT, GalNAc transferase; GFAT, glutamine‐fructose‐6‐phosphate transaminase; G0, G0 glycoform; GlcN‐6‐P, glucosamine‐6‐phosphate; GlcNAc, *N*‐acetylglucosamine; GlcNAc‐1‐P, *N*‐acetylglucosamine‐1‐phosphate; GlcNAc‐6‐P, *N*‐acetylglucosamine‐6‐phosphate; Glu, glutamate; Gln, glutamine; GnT1/2/4/5, glucosamine transferase 1/2/4/5; HK, hexokinase; MAN2, mannosidase 2; Man5, mannose‐5 glycoform; OGA, *O*‐GlcNAcase; OGT, O‐GlcNAc transferase; PFK, phosphofructokinase; UDP‐GalNAc, uridine diphosphate *N*‐acetylgalactosamine; UDP‐GlcNAc, uridine diphosphate *N*‐acetylglucosamine.

Glycosylation is a critical quality attribute that influences the immunogenicity, efficacy, and serum half‐life of therapeutic proteins. mAbs typically possess a single *N*‐glycosylation site on their Fc domain, whereas Fc‐fusion proteins can have more complex glycosylation profiles, featuring multiple *N*‐glycosylation and *O*‐glycosylation sites. The quantity, positioning, and structure of these glycan sites are determined by the specific partner proteins fused to the Fc part [6, 7]. N‐linked glycosylation initiates with the transfer of a preassembled Glc3Man9GlcNAc2 oligosaccharide from dolichol phosphate to an asparagine residue within the peptide chain. This initial glycan structure undergoes modification in the endoplasmic reticulum, resulting in the formation of a Man5GlcNAc2 (Man5 glycoform) oligosaccharide. Subsequently, this structure is further processed in the Golgi apparatus to yield a conserved core structure GlcNAc2Man3GlcNAc2 (G0 glycoform). Additional sugar residues, including fucose, galactose, and *N*‐acetylneuraminic acid, may be added to this core structure, leading to the formation of complex glycans [8]. In contrast to *N*‐glycosylation, mucin‐type *O*‐glycosylation – found in Fc‐fusion proteins such as Etanercept and Abatacept [6] – lacks a specific core structure. Mucin‐type *O*‐glycosylation begins with the transfer of an *N*‐acetylgalactosamine from uridine diphosphate *N*‐acetylgalactosamine (UDP‐GalNAc) onto a serine or threonine residue of a protein, which is then elongated by the addition of galactose, fucose, *N*‐acetylglucosamine (GlcNAc), and *N*‐acetylneuraminic acid [8]. Another form of *O*‐glycosylation, *O*‐GlcNAcylation, which is not present in Fc‐fusion proteins, involves the addition of a single GlcNAc from uridine diphosphate *N*‐acetylglucosamine (UDP‐GlcNAc) to a serine or threonine residue of a protein. The transfer of GlcNAc is catalyzed by O‐GlcNAc transferase and the removal of the O‐linked GlcNAc is catalyzed by *O*‐GlcNAcase. *O*‐GlcNAcylation plays a crucial role in regulating the activity of various enzymes, similar to phosphorylation [9] (Figure 1). Therefore, UDP‐GlcNAc and UDP‐GalNAc are essential substrates for several different types of glycosylation. UDP‐GlcNAc is the end product of the hexosamine biosynthesis pathway (HBP), where the rate‐limiting step is the conversion of fructose‐6‐phosphate and glutamine (Gln) to glucosamine‐6‐phosphate, catalyzed by glutamine‐fructose‐6‐phosphate transaminase (GFAT). This compound is subsequently metabolized to UDP‐GlcNAc through multiple enzymatic conversions. Notably, UDP‐GlcNAc can be dynamically and reversibly converted to UDP‐GalNAc by UDP‐glucose 4‐epimerase (Figure 1). The activity of the HBP can be assessed by quantifying the *O*‐GlcNAcylation of total protein, thereby indirectly determining the availability of UDP‐GlcNAc [10, 11, 12, 13, 14].

Given the importance of glycosylation in ensuring the functionality and efficacy of therapeutic proteins, control and reproducibility of glycosylation patterns is essential during the development of bioprocesses. Microbial expression systems lack the necessary protein processing machinery for human‐like glycosylation, which is why mammalian cells are preferred host cells for producing antibody‐based therapeutics. Among mammalian cell lines, Chinese Hamster Ovary (CHO) cells are the most commonly used cells for the industrial production of mAbs and Fc‐fusion proteins [6, 8, 15]. Of the aforementioned 19 Fc‐fusion proteins approved by the FDA by the end of 2025, at least 12 are produced using CHO cells [1]. In addition to selecting a suitable expression system, process parameters such as temperature, dissolved oxygen, and pH can also affect the glycan structure of a therapeutic protein [7]. Furthermore, the composition of cell culture media (CCM) presents another approach for modulating glycosylation.

Serum‐free CCM for industrial cell culture processes using CHO cells are complex mixtures often containing more than 50 components. Basic but important nutrients within CCM include salts, such as sodium, potassium, magnesium, calcium, chloride, phosphate, (bi)carbonate, sulfate, and nitrate. Sodium and potassium are especially important, as their ion gradients are essential for maintaining the transmembrane potential of cells. Therefore, CCM typically have sodium‐to‐potassium (Na:K) ratios ranging from 20:1 to 40:1. Additionally, salts are crucial for adjusting the osmolality of CCM to closely mimic the physiological conditions of cells, with CHO CCM generally maintaining an osmolality between 250 and 350 mOsmol/kg [16, 17]. Several studies have investigated the effects of increasing osmolalities through addition of NaCl on growth, titer, and recombinant protein quality of CHO cells. Pacis et al. found that elevated osmolalities resulted in higher levels of Man5 glycoforms in a mAb produced by CHO cells [18]. Similarly, Lee et al. observed a significant decrease in the proportion of highly sialylated and tetra‐antennary N‐linked glycans on an Fc‐fusion protein with increasing osmolality [19]. Additionally, Qin et al. reported lower ratios of galactosylation of glycans and a higher Man5 content in a mAb when osmolality was increased during a CHO fed‐batch process [20].

**FIGURE 2:**
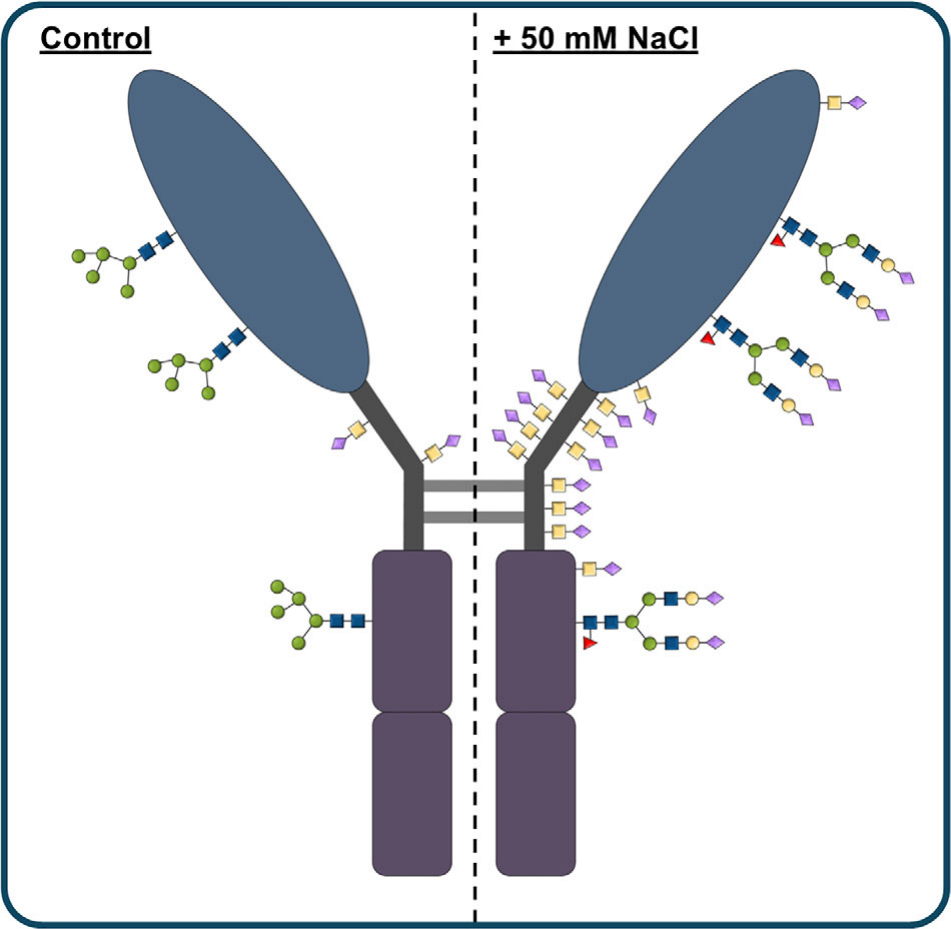
Effect of NaCl supplementation on the glycosylation of the investigated Fc‐fusion protein. The specific glycan positioning and structures are not intended to accurately reflect the actual fusion protein or its glycosylation patterns. This figure highlights the overall effects of enhanced glycosylation following NaCl supplementation, as observed in the previous studies by Bohl et al. [21].

A previous study in our group revealed an osmolalityindependent improvement in the glycosylation of an Fc‐fusion protein upon NaCl supplementation. A complex Fc‐fusion protein with multiple *O*‐glycosylation sites was investigated and a large ratio of low molecular weight (LMW) proteoforms were observed when the CHO cell line was cultivated in commercially available CHO medium. These LMW proteoforms were characterized as Fc‐fusion protein with less complex *N*‐glycans, exhibiting a higher proportion of terminal mannose and galactose, alongside a reduction in terminal GlcNAc and sialic acid, as well as considerably lower *O*‐glycan site occupancy. A study by Biel et al. [21] has previously shown that differences in *O*‐glycosylation can have an effect on the tumor necrosis factor‐α binding affinity of etanercept, highlighting that *O*‐glycosylation can impact the efficacy of therapeutic proteins [22]. Supplementation of the medium with NaCl improved the glycosylation of the Fc‐fusion protein drastically and the effect was dose‐dependent with 50 mM yielding the highest *O*‐glycan site occupancy (Figure 2). In contrast, supplementation of (4S)‐2‐Methyl‐3,4,5,6‐tetrahydropyrimidine‐4‐carboxylic acid (ectoine), a commonly used osmolality control, did not improve the glycosylation of the Fc‐fusion protein. One expectation was that the increased availability of sodium or chloride ions might improve the synthesis of nucleotide sugars, the building blocks required for glycosylation [21].

The aim of this study was to confirm that the improvement in glycosylation of the Fc‐fusion protein was independent from the osmolality increase and to investigate whether the improved glycosylation occurred due to the additional sodium ions, chloride ions, or a shifted Na:K ratio. The glycosylation of the investigated Fc‐fusion protein might be affected by various processes, such as glycosyltransferase activity, substrate availability, or enzyme localization. This study specifically focused on the impact of NaCl supplementation on the activity of the HBP and thus on the availability of substrates required for glycosylation, particularly UDP‐GlcNAc.

## 2 Results

**FIGURE 3:**
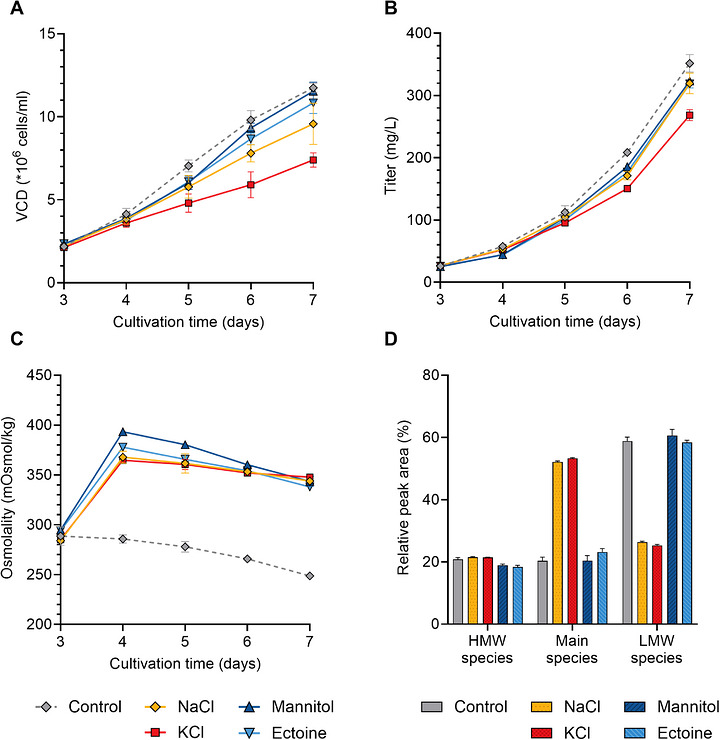
Impact of osmotically active substances on cell culture performance and product quality. Addition of different supplements (50 mM NaCl, 50 mM KCl, 100 mM ectoine, and 100 mM mannitol) on day 3 and their effect on cell growth (A) and recombinant protein titer (B). Relationship between the increase in osmolality of the cell culture medium (C) and the occurrence of LMW proteoforms (D). Error bars represent standard deviations (*n* = 3) of individual biological replicates. LMW, low molecular weight; HMW, high molecular weight.

In a 7‐day batch experiment, CHO cells producing an Fc‐fusion protein with complex glycosylation were cultivated using a commercially available CCM (control). On day 3, supplements were added to the medium. The addition of 100 mM of the osmolality controls – either ectoine or mannitol – resulted in cell growth comparable to the control condition. In contrast, the supplementation of 50 mM NaCl or KCl resulted in slightly lower viable cell densities (VCD) compared to the control (Figure 3A). All conditions, except for KCl supplementation, achieved final titers of approximately 320–360 mg/L by day 7, while the KCl‐supplemented condition yielded a lower final titer of 268 mg/L (Figure 3B). Supplementation of either of the salts or osmolality controls resulted in an increase in osmolality of about 100 mOsmol/kg compared to the control (Figure 3C). Cultivation of CHO cells in the commercially available medium resulted in a high ratio of LMW proteoforms (59%) of the Fc‐fusion protein. Although NaCl and KCl supplementation did not greatly affect cell growth or titer, the addition of these salts decreased LMW proteoforms by absolute 33%. This decrease appeared independently of the osmolality increase since the addition of ectoine or mannitol also raised osmolality without altering the LMW ratio (Figure 3D).

**FIGURE 4:**
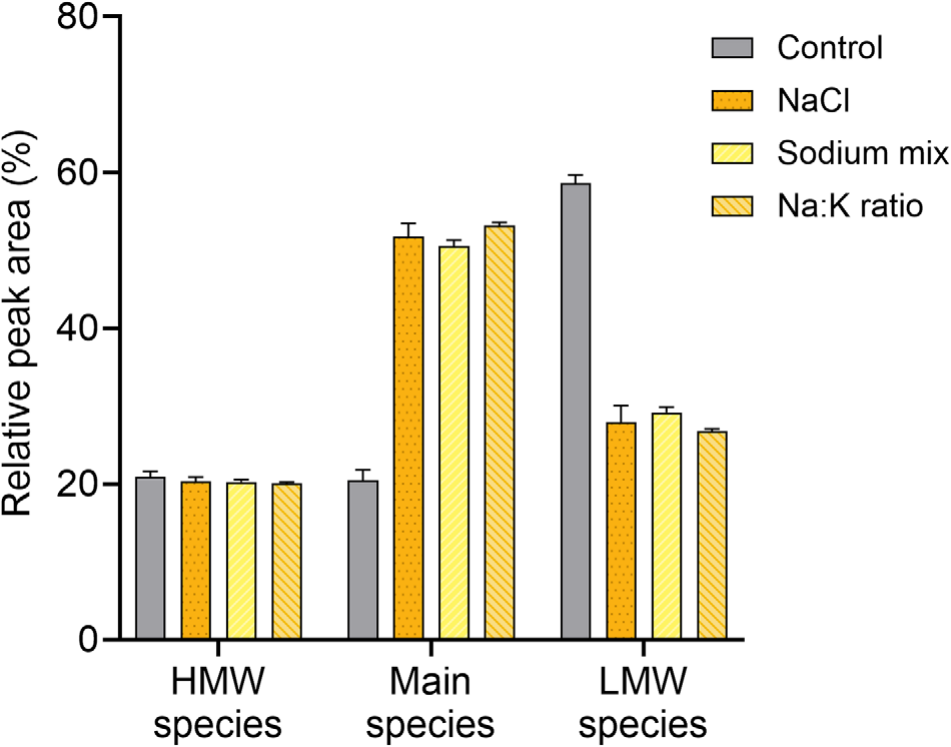
Decrease in low molecular weight proteoforms independently of chloride addition or sodium‐to‐potassium (Na:K) ratio adjustment. Size exclusion chromatography (SEC) results from a batch supplemented with a 50 mM sodium mix (4.2 mM sodium sulfate, 4.2 mM sodium acetate, 4.2 mM sodium bicarbonate, 4.2 mM disodium phosphate, 4.2 mM trisodium phosphate, and 4.2 mM trisodium citrate) and a batch where medium was supplemented with 50 mM NaCl and KCl, to obtain a comparable initial Na:K ratio as the control condition. Error bars represent standard deviations (*n* = 4) of individual biological replicates. LMW, low molecular weight; HMW, high molecular weight.

The addition of either 50 mM NaCl or KCl enhanced glycosylation of the Fc‐fusion protein, with both salts containing chloride ions. To test whether the LMW ratio was impacted by the increased sodium/potassium availability or by additional chloride, sodium supplementation without chloride was tested. Therefore, a mixture of sodium sources was prepared, containing 4.2 mM sodium sulfate, 4.2 mM sodium acetate, 4.2 mM sodium bicarbonate, 4.2 mM disodium phosphate, 4.2 mM trisodium phosphate, and 4.2 mM trisodium citrate, resulting in a total sodium concentration of 50 mM while minimizing the impact of each counterion. The sodium mix reduced the occurrence of LMW proteoforms similarly to the 50 mM NaCl supplementation, indicating that the improvement in glycosylation might be attributed to the additional sodium rather than chloride. Contrary to the 50 mM NaCl supplementation, the addition of the sodium mix increased the pH of the CCM from 6.5 to 7.2. The pH decreased back to 6.9 after about 3 h and back to pH 6.7 after 18 h. The increase in pH did not affect VCD or titer of the condition (Figure S1). Additionally, the supplementation of 50 mM NaCl led to an increase in the Na:K ratio of 10. To assess the effect of sodium concentration without altering the Na:K ratio, medium was supplemented with 50 mM NaCl and KCl, to obtain a comparable initial Na:K ratio as the control condition. This still led to a decrease in LMW proteoforms, suggesting that sodium availability is the main driver for improved glycosylation, rather than the adjusted Na:K ratio (Figure 4).

**FIGURE 5:**
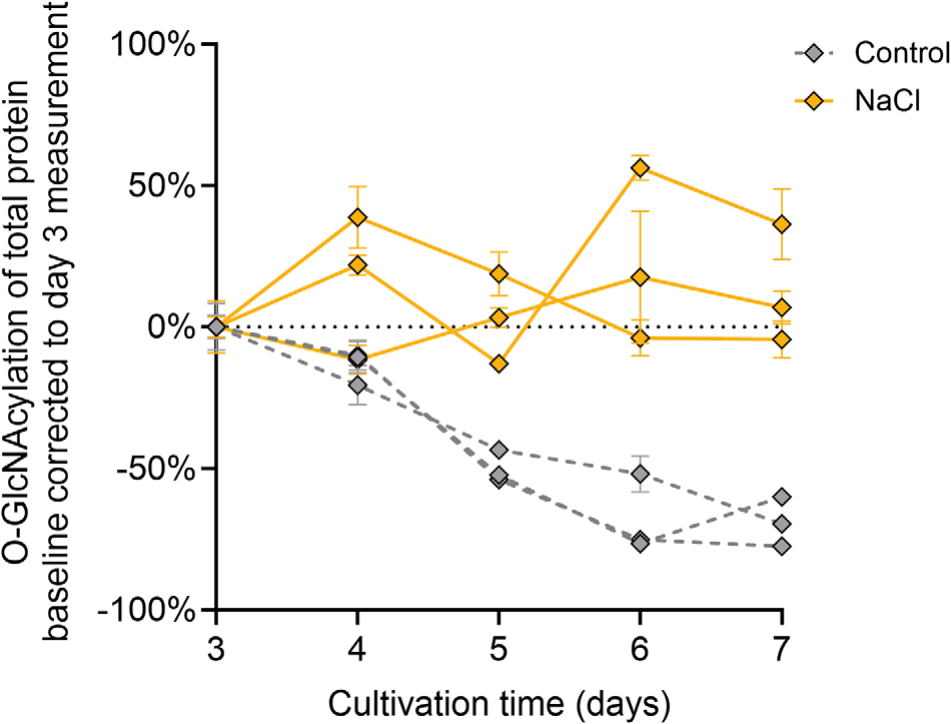
Measurement of *O*‐GlcNAcylation of total protein as an indicator of UDP‐GlcNAc availability and HBP activity. A capillary‐based Western Blot assay measured *O*‐GlcNAcylation of total protein in samples from three independent batch experiments. Error bars represent standard deviations (*n* = 2) of individual biological replicates.

Quantification of *O*‐GlcNAcylation of total protein from samples of the control condition and the medium supplemented with 50 mM NaCl in three independent experiments revealed a maintained level of *O*‐GlcNAcylated protein in the NaCl supplemented condition. *O*‐GlcNAcylation of total protein decreased over time in the control condition. This result indicates that the addition of NaCl maintained the activity of the HBP over time, leading to consistent availability of UDP‐GlcNAc compared to the control (Figure 5). Altered nucleotide sugar availability might be an explanation for the differences observed in glycosylation of the Fc‐fusion protein and thus the effects of precursor availability were investigated further.

**FIGURE 6:**
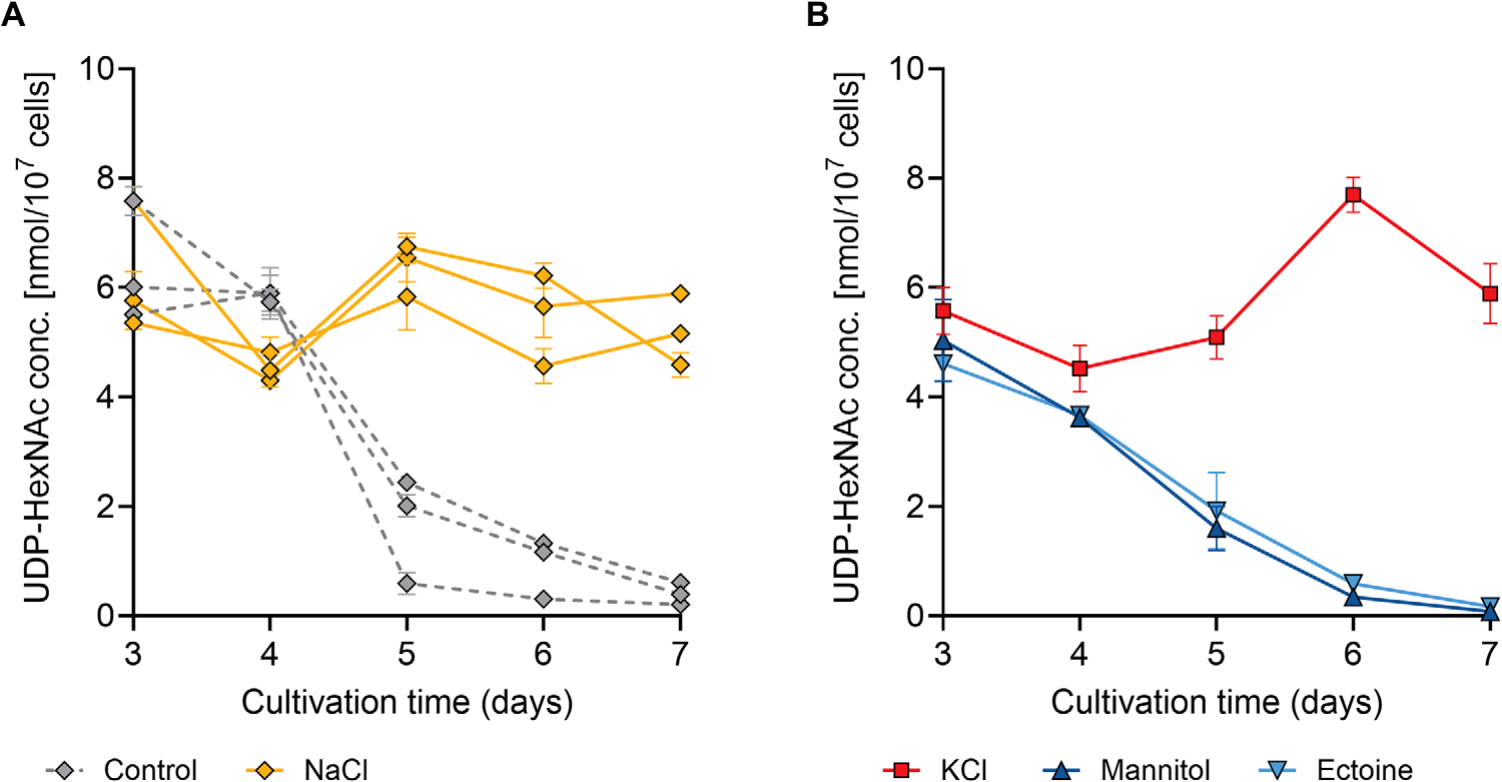
Quantification of combined UDP‐GlcNAc and UDP‐GalNAc (UDP‐HexNAc) concentration (intracellular) using a targeted LC‐MS/MS method. Measurement of control condition and NaCl condition of samples taken from three independent batch experiments (A) and KCl condition and osmolality controls from a single batch experiment (B). Error bars represent standard deviations (*n* = 2) of individual biological replicates.

Although the measurement of *O*‐GlcNAcylation of total protein serves as an indirect measurement of UDP‐GlcNAc availability, this measure might also be influenced by the abundance or activity of *O*‐GlcNAc transferase and *O*‐GlcNAcase, the enzymes responsible for *O*‐GlcNAcylation. To address this, the intracellular concentrations of UDP‐GlcNAc and UDP‐GalNAc were measured. However, the applied method did not allow for separate quantification of these two metabolites, leading to their combined measurement as UDP‐HexNAc. Nonetheless, since both activated sugars play a crucial role in the glycosylation of the Fc‐fusion protein and are dynamically and reversibly interconverted by UDP‐glucose‐4‐epimerase, their analysis as a sum parameter still provides valuable insights. Quantification of UDP‐HexNAc for the control condition initially showed a concentration of 6–8 nmol/10^7^ cells, with a considerable decline on day 5 and nearing a depletion in UDP‐HexNAc by day 7. In contrast, the supplementation of NaCl maintained a constant intracellular UDP‐HexNAc concentration throughout the cultivation time (Figure 6A). To demonstrate that the effects observed with NaCl supplementation on UDP‐HexNAc concentration were independent of osmolality, the UDP‐HexNAc levels were also assessed in the KCl supplemented condition, as well as in the two osmolality control conditions. The addition of KCl resulted in a relatively stable UDP‐HexNAc level throughout the batch experiment, comparable to the addition of NaCl. In contrast, supplementation with mannitol or ectoine resulted in a decline in UDP‐HexNAc concentrations similar to that observed in the control condition (Figure 6B). Notably, the trends in UDP‐HexNAc concentrations mirrored those of the LMW proteoforms under these conditions. Therefore, the supplementation of either NaCl or KCl appears to impact the availability of UDP‐HexNAc independently of osmolality changes.

**FIGURE 7:**
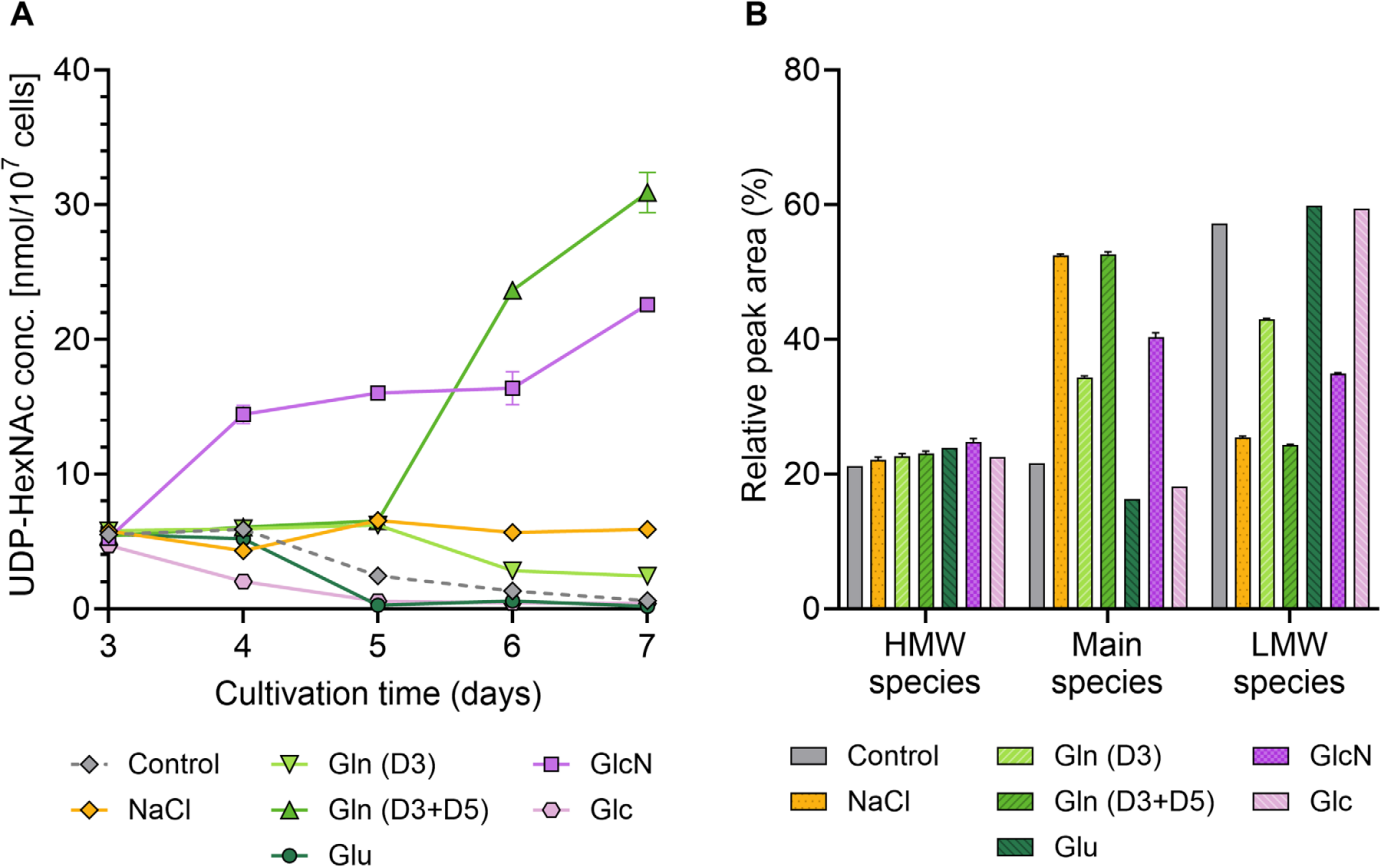
Impact of the supplementation of HBP substrates Gln, Glu, Glc, and GlcN on intracellular UDP‐HexNAc concentration and LMW proteoform occurrence. Medium was supplemented with 6 mM Glu, 6 mM Gln, 6 mM Glc or 1 mM GlcN on day 3 or with 6 mM Gln on day 3 and day 5. Intracellular UDP‐HexNAc concentration (A) was measured with a quantitative LC‐MS/MS method and LMW species abundance (B) was determined by SEC. Error bars represent standard deviations (*n* = 2) of individual biological replicates. D3, glutamine addition on day 3; D3 + D5, glutamine addition on day 3 and 5; Glc, glucose; GlcN, glucosamine; Gln, glutamine; Glu, glutamate; HMW, high molecular weight; LMW, low molecular weight.

To determine if the observed differences in UDP‐HexNAc availability are caused by altered HBP activity and to assess the connection between UDP‐HexNAc availability and the occurrence of LMW proteoforms, medium was supplemented with Gln – a substrate of GFAT, the rate‐limiting enzyme of the HBP – and glucosamine (GlcN), which is part of the hexosamine salvage pathway that generates UDP‐GlcNAc by bypassing GFAT (Figure 1). Additionally, glucose and glutamate supplementation were tested as controls. Glutamine and GlcN supplementation did not markedly affect VCD or titer compared to the control, whereas glutamate and glucose supplementation resulted in moderately lower VCDs throughout cultivation (Figure S2). Supplementation of NaCl led to a stable UDP‐HexNAc concentration around 6 nmol/10^7^ cells while the nucleotide sugar concentration declined toward a depletion in the control condition. Supplementation with 6 mM Gln on only day 3 led to a stable UDP‐HexNAc concentration up to day 5, followed by a decline on day 6. When Gln was supplemented on day 3 and day 5, a steep increase in UDP‐HexNAc was observed following the day 5 addition. Compared to the control, the addition of 1 mM GlcN on day 3 considerably increased UDP‐HexNAc concentration on days 4–6, with a further increase observed on day 7. Both the addition of glutamate and glucose led to a decline in UDP‐HexNAc concentration comparable to the control (Figure 7A). A single addition of Gln on day 3 resulted in a 14.2% decrease in LMW proteoforms. The additional Gln bolus on day 5 led to a 32.9% decrease in LMW proteoforms, which was comparable to the decrease obtained with 50 mM NaCl. GlcN supplementation resulted in a 22.3% decrease in LMW proteoforms. Glutamate and glucose supplementation, despite lower VCDs, did not affect UDP‐HexNAc concentration or LMW ratio compared to the control, highlighting that the changes are driven by HBP substrate availability rather than differences in cell density (Figure 7B). Overall, these results indicate a link between HBP substrate availability, the concentration of the nucleotide sugars UDP‐GlcNAc and UDP‐GalNAc and the occurrence of the LMW proteoforms.

## 3 Discussion

Glycosylation is a critical quality attribute for therapeutic mAbs and Fc‐fusion proteins and therefore an essential consideration during process development. Sodium concentration in CCM markedly influenced glycosylation of an Fc‑fusion protein and the effect of sodium availability on the HBP was investigated.

In a previous study performed in our group [21], a decrease in LMW proteoforms of an Fc‐fusion protein produced in CHO cells was observed when the medium was supplemented with NaCl. These LMW proteoforms were characterized as Fc‐fusion proteoforms with reduced glycosylation, for instance by having less complex *N*‐glycan structures and a severely reduced *O*‐glycan site occupancy. These results indicated that the addition of NaCl improved the glycosylation of the Fc‐fusion protein. Additionally, this effect was shown to be independent from the osmolality increase, since supplementation of an iso‐osmotic concentration of ectoine did not affect glycosylation. Ectoine was selected as the osmolality control due to its biocompatibility and its ability to be taken up by cells without interfering with their metabolism. Ectoine also acts as a protectant, shielding cells from harmful environmental conditions such as high temperatures or osmotic stress [23, 24]. For instance, ectoine reversed the growth inhibition caused by hyperosmolality in *Escherichia coli* [25]. Given ectoine's protective properties, it is important to re‐evaluate its suitability as an osmolality control. Indeed, the reduction of LMW proteoforms induced by NaCl might still be attributed to osmotic stress, which may not have been observed with iso‐osmotic ectoine supplementation due to ectoine's osmoprotectant properties. To further validate the independence of osmolality, mannitol was tested as another osmolality control. Mannitol is commonly found as an osmolality control in the literature, does not enter cells, is not an osmoprotectant and is considered one of the most neutral osmolytes [26, 27, 28]. Neither the supplementation of mannitol nor ectoine reduced the LMW ratio, reinforcing the conclusion that the change in glycosylation with NaCl supplementation is not caused by the increased osmolality. Although many studies investigate the effects of hyperosmolality on CHO cells through NaCl addition, they often fail to include non‐ionic osmolytes as controls [18, 19, 20, 29, 30, 31, 32, 33, 34]. This oversight can lead to misattributing observed effects to increased osmolality when they may actually be due to the additional sodium or chloride ions, as highlighted by the results of this study. Conversely, Jarusintanakorn et al. investigated the effects of adding 100 mM ectoine to CHO cells [35], resulting in an increase in productivity and cell volume as well as cell cycle arrest in G0/G1 phase. The addition of 100 mM ectoine to CCM increased osmolality by 100 mOsmol/kg, yet the authors attributed all findings solely to ectoine, overlooking the potential influence of the osmotic shift. In fact, hyperosmolality was often reported to result in increased productivity, increased cell volume and cell cycle arrest [29, 36]. Our study demonstrated an osmolality‐independent effect of NaCl on CHO cells, emphasizing the necessity of implementing appropriate osmotic controls. This approach is essential for accurately attributing observed outcomes to either osmotic shifts or the active substances, depending on the specific research question.

Given that the osmotic shift was ruled out as the cause of the observed differences in LMW occurrence, we hypothesized that the increased availability of sodium or chloride ions might influence glycosylation. The supplementation of 50 mM KCl, which introduces no additional sodium, also resulted in a decrease in the LMW ratio, prompting the initial assumption that chloride ions, rather than sodium, were primarily responsible for the observed improvements in glycosylation. *N*‐glycan branching occurs in the Golgi apparatus, where an acidic pH is crucial for its proper functioning. The Golgi pH regulator, a chloride channel, is essential for the continuous pumping of H^+^ ions by the V‐ATPase, thereby maintaining the acidic environment of the Golgi [37]. Maeda et al. reported that the abundance of this anion channel significantly affects the *N*‐ and *O*‐glycosylation of proteins and lipids [38]. We hypothesized that chloride availability influences Golgi pH regulator activity and, consequently, the glycosylation of the Fc‐fusion protein in this study. The supplementation of NaCl increased the availability of chloride ions, aiding in the maintenance of acidic Golgi pH and enhancing glycosylation. However, the fact that supplementation of a mix of sodium sources, which did not provide additional chloride, still led to a reduction in the LMW ratio, suggests that chloride ion availability may not be the primary driver of glycosylation improvement. Instead, it appears that the presence of monovalent cations, such as sodium or potassium, affect *O*‐glycan site occupancy and *N*‐glycan complexity.

The literature does not propose a direct link between sodium or potassium concentration and glycosylation. However, literature suggests that glycosylation can be affected by factors such as glycosyltransferase activity [7, 39] and the availability of nucleotide sugars [40]. In this study, a maintained intracellular concentration of UDP‐GlcNAc/UDP‐GalNAc over time was observed with the supplementation of NaCl or KCl while a decrease in these nucleotide sugars was observed for the control. This result indicates that the availability of sodium or potassium affected the activity of the HBP. One way in which sodium interacts with cells is through the coupled transport and uptake of nutrients, such as glucose and amino acids [41, 42, 43, 44, 45, 46]. A study by Oh et al. demonstrated that increasing the osmolality of the medium with NaCl resulted in an increase in intracellular amino acid concentrations. However, this enhanced amino acid uptake was not observed when osmolality was increased in the absence of sodium ions, indicating that sodium is integral to this process [47]. In particular, Gln, which serves as a substrate for GFAT, the rate‐limiting enzyme in the HBP, is of interest. GFAT catalyzes the conversion of Gln and fructose‐6‐phosphate into glutamate and glucosamine‐6‐phosphate. The latter is further processed through the HBP to produce UDP‐GlcNAc. Although the cell line used in this study was a glutamine synthetase (GS) cell line, implying that Gln was not present in the tested medium, the presence of additional sodium might have influenced the uptake of other amino acids. This may have led to an increase in the availability of intracellular Gln through metabolic processes, thereby raising Gln availability for the synthesis of UDP‐GlcNAc. Fructose‐6‐phosphate can also be converted by phosphofructokinase (PFK) into fructose‐1,6‐bisphosphate, which is subsequently processed and funneled into glycolysis for energy production. An increased uptake of amino acids may lead to the enhanced production of metabolic intermediates that feed into the tricarboxylic acid cycle. For instance, increased uptake of aspartate and asparagine may enhance the abundance of oxaloacetate, an intermediate in the tricarboxylic acid cycle. Aspartate can be converted to oxaloacetate through transamination, while asparagine can be deaminated to yield additional aspartate, further increasing oxaloacetate availability [48]. With a greater abundance of amino acids, the cells might produce more energy through anaplerosis, leaving more fructose‐6‐phosphate available to be funneled into the HBP via GFAT.

Sodium and potassium ions are crucial in generating and maintaining the resting membrane potential of cells. The addition of NaCl and KCl resulted in a decrease in LMW occurrence and the same was observed with the addition of a sodium mix not containing chloride, suggesting that the adjusted ion concentration of Na^+^ or K^+^ may influence the glycosylation differences of the Fc‐fusion protein. In the literature, increasing extracellular KCl concentration was found to depolarize the membrane potential of CHO cells [49]. Increasing extracellular potassium decreases the concentration gradient across the membrane and thus reduces the energy generated by the diffusive efflux of K^+^ ions through ion channels. To date, the impact of increasing extracellular NaCl on the membrane potential of CHO cells remains ambiguous as no existing studies definitively address this relationship. Miao et al. demonstrated that decreased sodium and chloride concentrations led to hyperpolarization in hamster vascular endothelial cells [50], implying that higher NaCl concentrations might induce depolarization. A higher level of extracellular NaCl increases the transmembrane sodium ion gradient. This enhanced concentration gradient facilitates the influx of Na^+^ through open sodium channels, resulting in membrane depolarization as the cell interior becomes more positively charged [51]. The impact of this influx of sodium ions might be particularly relevant for CHO cells as they have been reported to have the same ion conductance for sodium ions as for potassium ions [52] contrary to most other cells, which exhibit a greater conductivity for potassium ions [53, 54]. Although high sodium permeability suggests that NaCl supplementation might lead to membrane depolarization, CHO cells also exhibit high chloride conductance [52]. This chloride conductance might counteract the depolarizing effect of sodium influx as the supplementation of NaCl is expected to also lead to an increased influx of Cl^−^ ions. Ultimately, the specific effects of these salt supplementations on the membrane potential of this specific CHO cell line require experimental validation, as membrane conductance varies considerably between cell lines. Interestingly, nonproliferating cells, such as muscle and nerve cells, typically have resting membrane potentials ranging from −70 to −90 mV contrary to established, highly proliferating cell lines in tissue culture that tend to have a lower membrane potential [55]. For CHO cells, differing resting membrane potentials were reported such as −5 to −10 mV [56], −12 mV [52] or −37 mV [57]. The difference in cell lines is also evident when looking at the literature regarding the existence of voltage‐activated Ca^2+^ channels in CHO cells. Gamper et al. reported no detection of voltage‐gated calcium channels in CHO cells [56]. Meanwhile, Skryma et al. identified voltage‐dependent calcium channels in CHO cells but only in a fraction of the tested cells [58]. Similarly, Mitsuhashi et al. observed that membrane depolarization was accompanied by increased intracellular calcium [49]. Overall, the literature presents limited and sometimes contradictory data on how membrane potential in CHO cells is influenced by shifts in extracellular ion balance, particularly independent of osmolality changes. Assuming that increases in K^+^ or Na^+^ lead to membrane depolarization in CHO cells, voltage‐gated calcium channels might be activated leading to an increase in intracellular calcium. Calcium plays a vital role in cell signaling, and this hypothesized increase might have triggered a signaling cascade that activates GFAT and inhibits PFK. These enzyme regulations might enhance the flux of fructose‐6‐phosphate through the HBP, generating more UDP‐GlcNAc and, subsequently, more substrate for glycosylation of the Fc‐fusion protein. This increased glycosylation being reflected in the decrease in LMW proteoforms.

In conclusion, this study underscores the critical role of sodium and potassium ions in influencing the glycosylation of an Fc‐fusion protein, independently of osmotic effects. The findings suggest that the availability of these ions can considerably alter glycosylation patterns, potentially impacting the efficacy and safety of therapeutic proteins. By establishing the impact of sodium and potassium on glycosylation, the need for careful consideration of ion balance during CCM development becomes apparent. This research paves the way for more precise process development in the biopharmaceutical industry. Future studies might focus on determining the uptake of calcium, amino acids, and other nutrients. Furthermore, the regulation of GFAT and PFK may be explored to uncover the signaling pathways involved and to investigate the broader implications of ion concentration on cell metabolism and protein quality attributes.

## 4 Material and Methods

### 4.1 Chemicals and Reagents

The investigated CCM was chemically defined, and both the medium and all raw materials were purchased from Merck KGaA, Darmstadt, Germany. The CHO clone that was investigated in this study was developed through the random integration of a transgene into a suspension GS‐/‐ CHOZN host cell line. This specific cell line was established using traditional cell line development methods, including the GS system and single cell cloning. Uridine‐5′‐diphospho‐*N*‐acetylgalactosamine disodium salt (U5252) and Uridine‐5′‐diphospho‐*N*‐acetylglucosamine sodium salt (U4375) were obtained from Sigma‐Aldrich (Schnelldorf, Germany). Water (LC‐MS grade, 1.03728), acetonitrile (LC‐MS grade, 1.00029), methanol (LC‐MS grade, 1.06035), formic acid (LC‐MS grade, 5.33002) and ammonia solution (25%, LC‐MS grade, 5.33003) were purchased from Merck KGaA (Darmstadt, Germany).

### 4.2 Cell Culture and Spent media Analysis

Recombinant suspension CHOZN cells were passaged with a 2/2/3 subculture interval with seeding densities of 3*10^5^, 3*10^5^ and 2*10^5^ cells/mL, respectively. The cells were cultivated in spin tubes with vented caps (TPP, Techno Plastic Products AG) at 37°C, 5% CO_2_, 80% humidity and 230 rpm in a shake incubator (Kühner). This cell line produced an Fc‐fusion protein with complex glycosylation, specifically with many mucin‐type *O*‐glycosylation sites per monomer. For the batch experiments, cells were seeded at 2*10^5^ or 3*10^5^ cells/mL and cultivated over a 7‐day period. Samples were taken every day from day 3 onward. Supplements were always added as a stock solution on day 3, after all tubes had already been sampled. In contrast to all other conditions, for the 6 mM Gln supplementation, an additional bolus on day 5 was tested. VCD and viability were measured by flow cytometry (iQue3, Sartorius). Titer and other metabolites were monitored using a CEDEX BIO HT (Roche). Supernatant samples were utilized for recombinant protein purification, while cell pellets were washed twice with 1x PBS and flash‐frozen in liquid nitrogen for capillary Western Blot analysis and UDP‐HexNAc quantification. Osmolality was determined using the OsmoTECH Pro (Advanced Instruments).

### 4.3 Recombinant Protein Purification

Supernatant samples taken on day 7 were purified using Protein A PhyTips (PhyNexus Inc., PTR91‐40‐01) with RAININ E4 XLS and Pure Speed Protocol (Mettler Toledo, J1413990U). The PhyTips were equilibrated with 1x PBS pH 7.4. After protein capture (10 µg protein/µL Protein A resin), PhyTips were washed in 1x PBS pH 7.4 followed by a wash step with 140 mM NaCl. The proteins were eluted using 30 mM citric acid at pH 3.0 and immediately neutralized with 0.375 M Tris base at pH 9.0.

### 4.4 Size Exclusion Chromatography

Separation was performed isocratically (0.35 mL/min) at room temperature using an Acquity UPLC H class (Waters) with software Empower 3. Samples first passed a TSKgel guard (4.6 mm i.d × 3.5 cm, 4 µm) (Supelco 818762) which served as a pre‐column followed by separation on TSKgel Super SW 3000 Column (4.6 mm i.d × 30 cm) (Tosoh; 0018675). The mobile phase consisted of 50 mM sodium phosphate and 400 mM sodium perchlorate at pH 6.3. Proteins passed a 500 µm titanium flow cell (Waters) and were subsequently detected at 214 nm.

### 4.5 *O*‐GlcNAcylation of Total Protein

Cell pellets were lysed in RIPA lysis buffer containing 1% SDS and 1X protease and phosphatase inhibitor cocktail (Thermo Fisher Scientific, 78440). Protein concentrations were assessed with the Pierce Detergent Compatible Bradford Assay Kit (Thermo Fisher Scientific, 23246). *O*‐GlcNAcylation of total protein was measured by Capillary Western Blot using a Jess device (ProteinSimple) with the 12–230 kDa separation module (Biotechne, SM‐W001), following the manufacturer's specifications. An anti‐O‐linked *N*‐Acetylglucosamine antibody (Abcam, ab2739) was used alongside an antimouse secondary horseradish peroxidase antibody (ProteinSimple, 042–205). Following the O‐linked GlcNAc immunoassay, antibodies were removed using the RePlex module to run a subsequent total protein detection for normalization. Data analysis was performed using Compass software version 7.0.0.

### 4.6 Sample Preparation and LC‐MS/MS Analysis of UDP‐HexNAc

For metabolite extraction, ice cold methanol/water (75/25, v/v) was added to the cell pellets, followed by homogenization using a thermomixer for 30 min (1600 rpm, 4°C). After centrifugation (9000 g, 5 min), the supernatants were dried using a vacuum centrifuge (1400 rpm, 45°C, 20 mbar) and reconstituted in water/acetonitrile (95/5, v/v). LC‐MS/MS measurements were done using an Agilent 1290 Infinity II instrument coupled to an Agilent 6495C triple quadrupole mass spectrometer with an electrospray ion source in negative ionization mode.

Separation was performed on a Supel Carbon LC HPLC column (2.7 µm, 10 cm 0× 3.0 mm; Merck KGaA, 59993‐U) with an injection volume of 20 µL at a flow rate of 0.70 mL/min over a period of 20 min using solvent A (water + 0.1% formic acid, adjusted to pH 9.0 with NH_4_OH) and solvent B (acetonitrile) as mobile phases. Elution was performed at a column oven temperature of 50°C using a gradient from 5% to 12% B in 1 min, 12% B for 2 min, 12% B to 45% in 4 min, 45% B to 95% in 3 min, 95% B for 5 min, returning to 5% B in 0.5 min and re‐equilibration at 5% B for 4.5 min. LC‐MS parameters are shown in the Supplemental Data (Table S1). Data was evaluated using the MassHunter Software “Quantitative Analysis for QQQ” from Agilent Technologies. Quantification was performed by means of an external calibration in a calibration range of 0.5–250 ng/mL. A mixture of UDP‐GlcNAc and UDP‐GalNAc in a 1:1 ratio was used to prepare the calibration solutions.

### 4.7 Use of Artificial Intelligence

Artificial Intelligence Generated Content tools (GPT‐4o mini) and large language models were used solely to enhance language, grammar, and clarity. All scientific content was generated by the authors.

## Author Contributions

**Jason Reiniger**: Conceptualization, methodology, investigation, visualization, writing – original draft preparation. **Adrian Pöttner**: Investigation, writing – review and editing. **Lara Rosenberger**: Methodology, investigation, writing – review and editing. **Aline Zimmer**: Supervision, project administration, writing – review and editing.

## Funding

This study was funded by Merck Life Science KGaA.

## Conflicts of Interest

Authors J.R., A.P. and A.Z. are employed by the company Merck Life Science KGaA. Author L.R. is employed by the company Merck KGaA.

## Supporting information




**Supporting File**: biot70261‐sup‐0001‐SuppMat.docx.

## Data Availability

The data that support the findings of this study are available from the corresponding author upon reasonable request. Raw data are available from the corresponding author upon request.
