# Supplementary material for: Osmolality‐Independent Impact of Sodium on Glycosylation of an Fc‐Fusion Protein and the Hexosamine Biosynthesis Pathway in a Chinese Hamster Ovary Cell Line
Source: Biotechnol J. 2026 Jun 17;21(6):e70261. doi: 10.1002/biot.70261 (PMC13274331; doi:10.1002/biot.70261)
Supplement: Supplementary file 1 — Supporting File: biot70261‐sup‐0001‐SuppMat.docx. [file BIOT-21-e70261-s001.docx]

# Supplemental Information


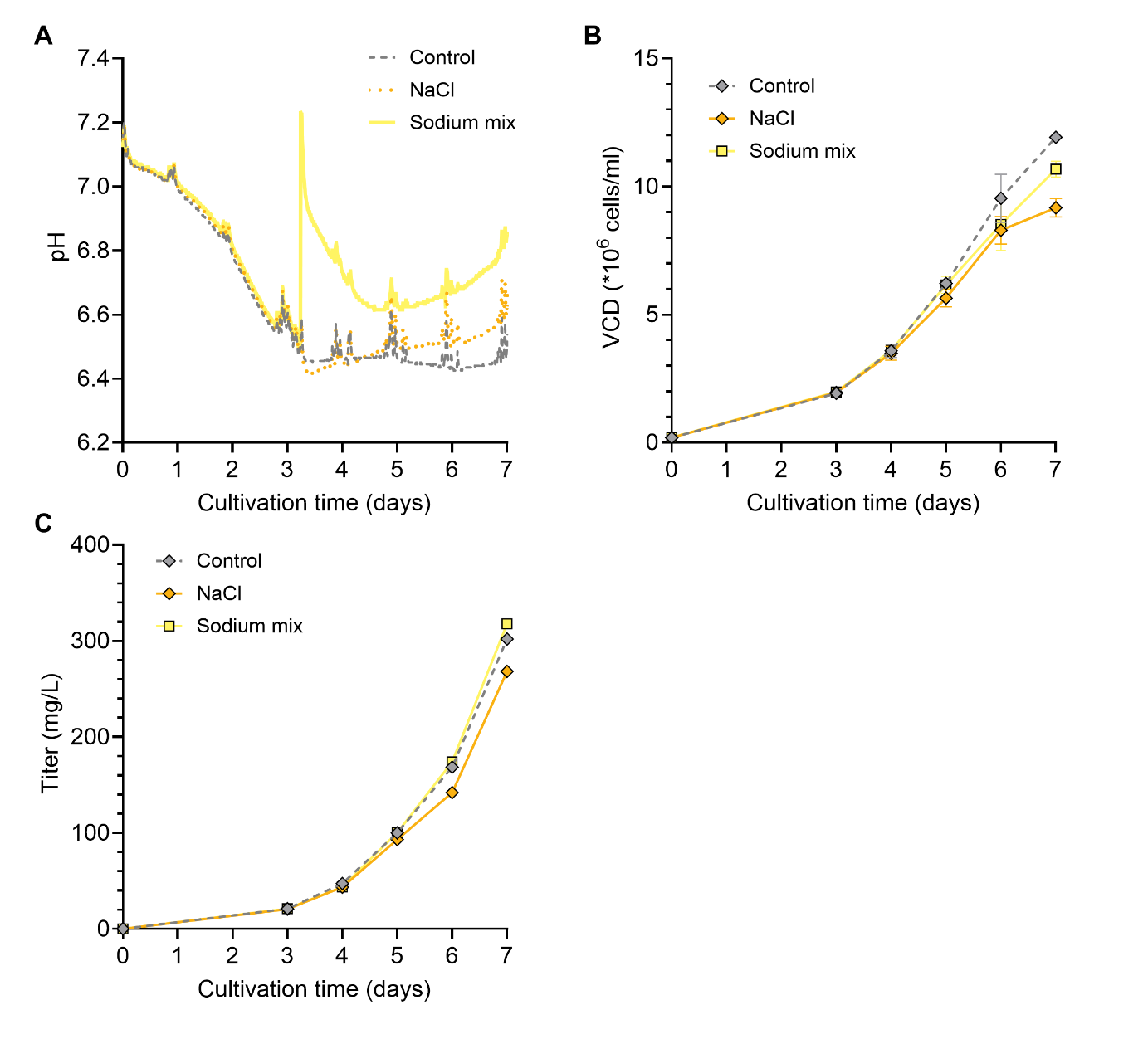


Supp. Figure 1: Impact of sodium mix supplementation on pH of the CCM (A), cell growth (B) and titer (C). Online measurement of pH in the CCM using an ITR iTube96Reader (PreSens).


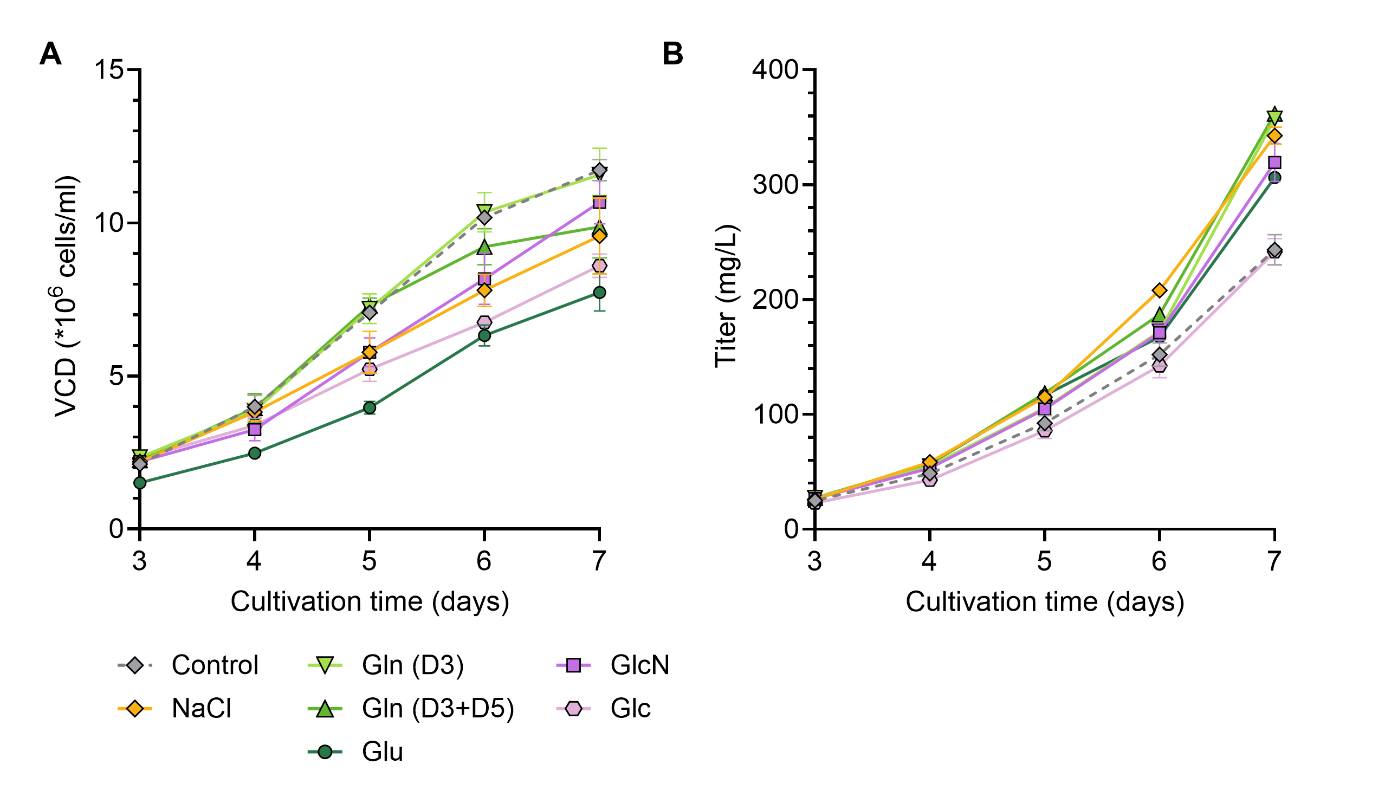


Supp. Figure 2: Impact of the supplementation of HBP substrates Gln, Glu, Glc and GlcN on VCD (A) and titer (B). Medium was supplemented with 6 mM Glu, 6 mM Gln, 6 mM Glc or 1 mM GlcN on day 3 or with 6 mM Gln on day 3 and day 5. Error bars represent standard deviations (n = 4) of individual biological replicates. D3, glutamine addition on day 3; D3+D5, glutamine addition on day 3 and 5; Glc, glucose; GlcN, glucosamine; Gln, glutamine; Glu, glutamate; HMW, high molecular weight; LMW, low molecular weight.

Supp. Table 1: LC-MS conditions for the analysis

| **LC-MS instrument** | | 1290 Infinity II instrument coupled to an 6495C triple quadrupole mass spectrometer (Agilent Technologies) | | | | | |
| --- | --- | --- | --- | --- | --- | --- | --- |
| **Column** | | Supel™ Carbon LC HPLC column; 10 cm x 3.0 mm, 2.7 µm (Merck KGaA) | | | | | |
| **Column temperature [°C]** | | 50 | | | | | |
| **Mobile phase** | | Water + 0.1% formic acid (adjusted to pH 9 with NH_4_OH) (**A**),  Acetonitrile (**B**) | | | | | |
| **Gradient** | | Time [min] | | **A** [%] | | **B** [%] | |
|  |  | 0.0 | | 95.0 | | 5.0 | |
|  |  | 1.0 | | 88.0 | | 12.0 | |
|  |  | 3.0 | | 88.0 | | 12.0 | |
|  |  | 7.0 | | 55.0 | | 45.0 | |
|  |  | 10.0 | | 5.0 | | 95.0 | |
|  |  | 15.0 | | 5.0 | | 95.0 | |
|  |  | 15.5 | | 95.0 | | 5.0 | |
|  |  | 20.0 | | 95.0 | | 5.0 | |
| **Flow rate [ml/min]** | | 0.70 | | | | | |
| **Injection volume [µl]** | | 20.0 | | | | | |
| **Acquisition & data evaluation software** | | MassHunter Workstation: LC/MS Data acquisition and Quantitative Analysis for QQQ Version 10.1 (Agilent Technologies) | | | | | |
| **Ionisation mode (polarity)** | | ESI (negative) | | | | | |
| **Acquisition mode** | | Multiple Reaction Monitoring | | | | | |
| **Gas temperature [°C]** | | 250 | | | | | |
| **Gas flow [L/min]** | | 11 | | | | | |
| **Nebulizer [psi]** | | 25 | | | | | |
| **Sheat gas temperature [°C]** | | 400 | | | | | |
| **Sheat gas flow [L/min]** | | 12 | | | | | |
| **Capillary voltage (negative) [V]** | | 2000 | | | | | |
| **Nozzle voltage (negative) [V]** | | 0 | | | | | |
| **High Pressure RF (negative) [V]** | | 90 | | | | | |
| **Low Pressure RF (negative) [V]** | | 60 | | | | | |
| **Multiple Reaction Monitoring transitions of UDP-GalNAc/UDP-GlcNAc (Quantifier/Qualifier*):** | | | | | | | |
| **Analyte** | **Compound** | | **Precursor ion** **[*m/z*]** | | **Product ion** **[*m/z*]** | | **CE** **[V]** |
| 1 | UDP-GalNAc/UDP-GlcNAc | | 606.0 | | 79.0 | | 80 |
|  | UDP-GalNAc/UDP-GlcNAc* | | 606.0 | | 272.9 | | 40 |
